# Viral immunogenicity determines epidemiological fitness in a cohort of DENV-1 infection in Brazil

**DOI:** 10.1371/journal.pntd.0006525

**Published:** 2018-05-29

**Authors:** Tauyne Menegaldo Pinheiro, Mânlio Tasso de Oliveira Mota, Aripuanã Sakurada Aranha Watanabe, Joice Matos Biselli-Périco, Betânia Paiva Drumond, Milene Rocha Ribeiro, Danila Vedovello, João Pessoa Araújo, Paulo Filemon Paolucci Pimenta, Bárbara Aparecida Chaves, Mayara Marques Carneiro da Silva, Izabella Cristina Andrade Batista, Michelle Premazzi Papa, Lana Monteiro Meuren, Carolina Gonçalves de Oliveira Lucas, Flavio Lemos Matassoli, Laura Helena Vega Gonzales Gil, Adriana Bozzi, Carlos Eduardo Calzavara-Silva, Luciana Barros de Arruda, Danielle da Glória de Souza, Mauro Martins Teixeira, Nikos Vasilakis, Maurício Lacerda Nogueira

**Affiliations:** 1 Laboratório de Pesquisa em Virologia, Departamento de Doenças Dermatológicas, Infecciosas e Parasitárias, Faculdade de Medicina de São José do Rio Preto, SP, Brazil; 2 Laboratório de Vírus, Instituto de Ciências Biológicas, Universidade Federal de Minas Gerais, Belo Horizonte, MG, Brazil; 3 Laboratório de Virologia, Departamento de Microbiologia e Imunologia, Instituto de Biociências, Universidade Estadual Paulista Júlio de Mesquita Filho, Botucatu, SP, Brazil; 4 Laboratório de Entomologia Médica, Centro de Pesquisas René Rachou, Fiocruz, Belo Horizonte, MG, Brazil; 5 Programa de Pós-Graduação em Medicina Tropical, Universidade do Estado do Amazonas (UEA) e Fundação de Medicina Tropical Dr. Heitor Vieira Dourado (FMT-HVD), Manaus, AM, Brazil; 6 Departamento de Virologia, Centro de Pesquisas Aggeu Magalhães, Fiocruz, Recife, PE, Brazil; 7 Laboratório de Imunologia Celular e Molecular, Centro de Pesquisas René Rachou, Fiocruz, Belo Horizonte, MG, Brazil; 8 Laboratório de Genética e Imunologia das Infecções Virais, Departamento de Virologia, Instituto de Microbiologia Prof. Paulo de Góes, Universidade Federal do Rio de Janeiro, RJ, Brazil; 9 Departamento de Microbiologia, Instituto de Ciências Biológicas, Universidade Federal de Minas Gerais, Belo Horizonte, MG, Brazil; 10 Laboratório de Imunofarmacologia, Departamento de Bioquímica e Imunologia, Instituto de Ciências Biológicas, Universidade Federal de Minas Gerais, Belo Horizonte, MG, Brazil; 11 Department of Pathology, Center for Biodefense and Emerging Infectious Diseases, Institute of Human Infections and Immunity, Center for Tropical Diseases, University of Texas Medical Branch, Galveston, United States of America; Oregon Health and Science University, UNITED STATES

## Abstract

The dynamics of dengue virus (DENV) circulation depends on serotype, genotype and lineage replacement and turnover. In São José do Rio Preto, Brazil, we observed that the L6 lineage of DENV-1 (genotype V) remained the dominant circulating lineage even after the introduction of the L1 lineage. We investigated viral fitness and immunogenicity of the L1 and L6 lineages and which factors interfered with the dynamics of DENV epidemics. The results showed a more efficient replicative fitness of L1 over L6 in mosquitoes and in human and non-human primate cell lines. Infections by the L6 lineage were associated with reduced antigenicity, weak B and T cell stimulation and weak host immune system interactions, which were associated with higher viremia. Our data, therefore, demonstrate that reduced viral immunogenicity and consequent greater viremia determined the increased epidemiological fitness of DENV-1 L6 lineage in São José do Rio Preto.

## Author summary

Since 2008 L6 is the endemic lineage that has circulated in SJRP. In 2010, the L1 lineage was first identified in the city. For a period, both lineages co-circulated, and then in 2013, L1 began to diminish until it was no longer detected in the population. Differences in replicative fitness are usually the main factor for clade replacement (CR) in DENV epidemics. However, despite the better viral fitness of the emerging lineage, the absence of CR could not be explained by these differences alone. Here, we combine epidemiological, phylogenetic, molecular and immunological analyses to provide a more precise understanding of the role of fitness in lineage dynamics with the persistence of L6 even after the introduction of L1 without CR. Differences in immune responses elicited by DENV-1 L1 and L6 lineages (genotype V), but not viral fitness in mosquito or human cells, explain the dynamics of circulating DENV in a city of Brazil.

## Introduction

The spread of dengue (DENV) over the past several decades has made this arbovirus infection a major public health concern of global impact [1,2]. The disease has a complex epidemiological pattern and a high economic impact globally and is considered hyperendemic, *i*. *e*. dengue fever has a high incidence and/or prevalence rate affecting all groups equally [3,4]. Worldwide, it is estimated that 390 million new DENV infections occur annually [1], and this number will likely increase with the creation of new vector habitats due to climate change [5]. Because of their wide distribution, particularly in urban and peri-urban environments in tropical and subtropical regions, mosquitoes of the *Aedes* genus are the main vectors of this disease [6,7]. DENV can lead to a wide spectrum of clinical manifestations that are classified by the World Health Organization (WHO) as *dengue without warning signs*, *dengue with warning signs* and *severe dengue* [8]. Previous infections with a heterologous type are usually, but not exclusively, associated with progression to more severe disease [1].

There are four genetically distinct serotypes of DENV (DENV-1 to -4) that share similar epidemiological features [6]. Each serotype is divided into distinct groups defined as genotypes, which in turn subdivided in different lineages [9,10]. The circulation of the virus is characterized by frequent lineage turnover, in which a lineage of circulating viruses is usually replaced by a new lineage, a well-documented phenomenon known as clade replacement (CR). CR can lead to an increase in the number of cases and in the severity of the disease [10–17]. In previous CR events, an established lineage circulating for a number of years in a given population is replaced when a new lineage is introduced. This replacement is usually followed by the extinction of the previous lineage after a period of co-circulation of both lineages [10–17].

Since the mid-1980s, DENV-1 has been circulating in Brazil. All Brazilian DENV-1 isolates described to date belong to genotype V, which is subdivided into three distinct clades (lineages 1, 3 and 6) [18–20]. These lineages were introduced into Brazil at different times, and CR or the co-circulation of different lineages has been observed in the country [18–20]. This pattern of co-circulation of different lineages was also observed in São José do Rio Preto (SJRP), São Paulo, Brazil.

Here, we combine phylogenetic, molecular and immunological analyses to describe the epidemiological dynamics of two Brazilian DENV-1 lineages (L1 and L6) circulating in SJRP from 2008 to 2015 to provide a more precise understanding of the role of fitness in lineage dynamics with the persistence of L6 even after the introduction of L1 without CR.

The reduced immunogenicity of L6, which contributed to B and T cell-specific immune response evasion, appears to have played a prominent role in its dominance over time.

## Results

### Phylogenetic analysis

**Fig 1:**
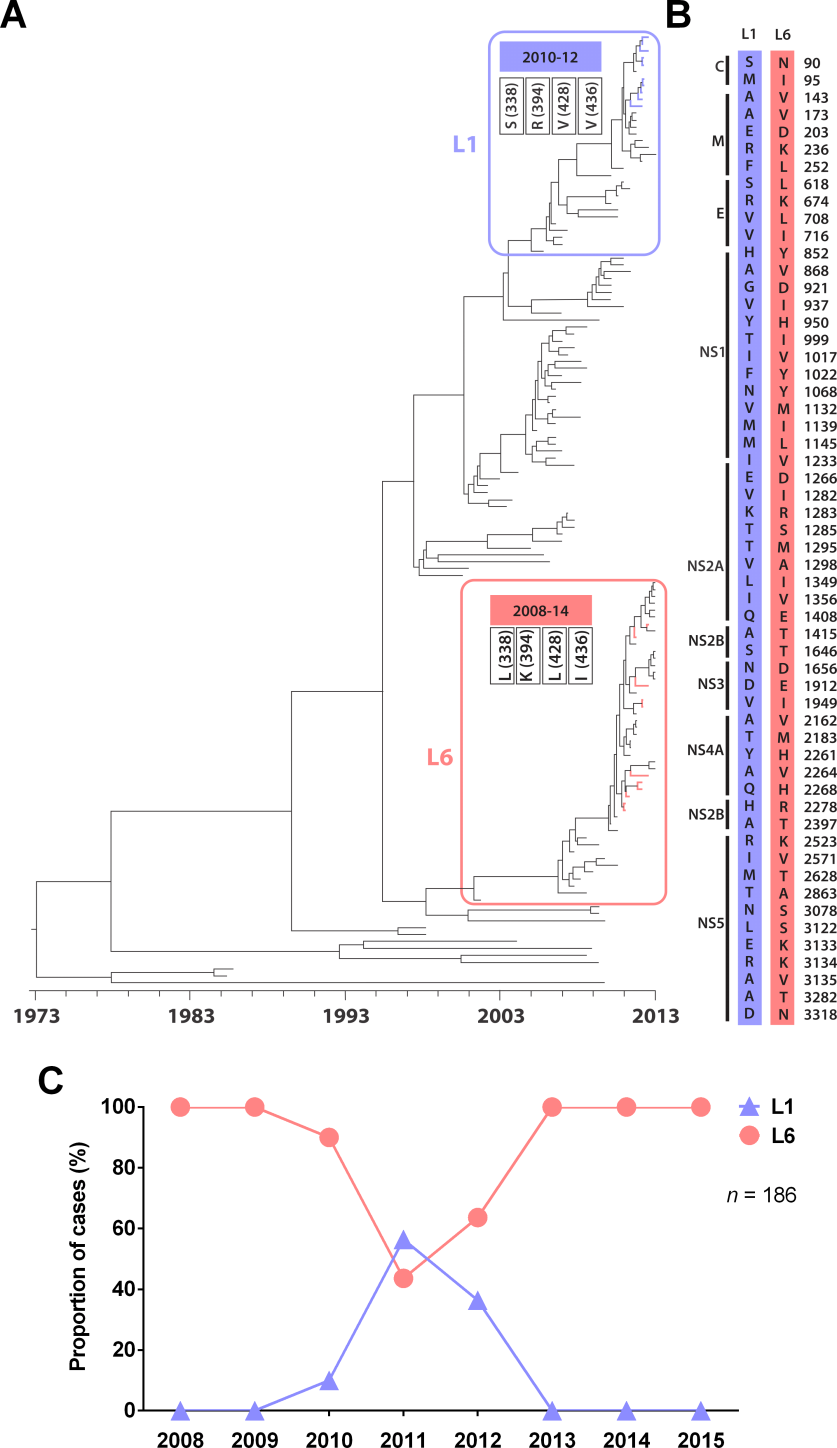
Evolutionary relationship between DENV-1 isolates and co-circulation of the two lineages from SJRP. (A) Phylogenetic tree of DENV-1 after Bayesian inference based on envelope nucleotide sequences with aa substitutions, characterizing the L1 and L6 lineages from SJRP, which are shown in blue and red, respectively. (B) Comparison of amino acid substitutions between the representative complete genome sequences of L1 and L6 lineages from SJRP (287/2011 and 484/2012, respectively). (C) Circulation of DENV-1 lineages (L1 and L6) in SJRP from 2008 to 2015 based on sequencing and genotyping data.

We used 20 envelope sequences (1,485 nucleotides) of DENV-1 isolates obtained from patients in SJRP, from 2008 to 2012, for the phylogenetic analyses. The results indicated that the isolates were subdivided into two lineages within genotype V. Isolates 59/2011, 287/2011, 354/2011, 395/2011, 422/2011, 430/2011, 437/2011, 442/2011, 492/2012 and 516/2012 were grouped within one lineage previously called L1 or lineage II [13,18] the most recent common ancestor (MRCA) for those species dates back to approximately 2008 (95% BCI = 2006–2009). Ten isolates from SJRP were grouped in another lineage, previously called L6 or lineage I [13,18]: 365/2008, 09/2009, 88/2010, 64/2011, 205/2011, 384/2011, 387/2011, 484/2012, 531/2012 and 552/2012. These isolates share an MRCA from approximately 2007 (2006–2008) (Fig 1A).

When deduced protein envelope sequences were analyzed, a total of four amino acid (aa) substitutions were observed between the two groups of isolates from SJRP. Those aa substitutions were observed at positions 338, 394 (located in domain III), 428 and 436 (located in the stem loop region). The aa observed in SJRP isolate sequences within L1 were as follows: 338, serine; 394, arginine; 428, valine; and 436, valine; whereas SJRP isolates within L6 presented leucine, lysine, leucine and isoleucine residues, respectively. A comparison between the complete genome sequences of the L1 and L6 lineages from SJRP (287/2011 and 484/2012, respectively) revealed 56 aa differences (Fig 1B).

The L6 lineage was first identified in SJRP in 2008, and the L1 lineage was detected only in 2010. These two lineages of DENV-1, genotype V, co-circulated in SJRP from 2010 until 2012. Based on sequencing or genotyping analysis by Taqman-based qPCR, 64 serum samples were identified as infected by L1 from 2010 to 2012, whereas 102 samples obtained from 2008 to 2015 were identified as L6, resulting in a total of 166 discriminated samples. In SJRP, L6 became the dominant circulating lineage after 2013 (Fig 1C).

### Viral fitness in viral dominance

#### Replicative fitness in vitro

To determine whether better viral fitness in the L6 lineage could explain its dominance in our study area, we assessed the viral replication of L1 and L6 isolates *in vitro*.

Initially, we tested two strains of each lineage and compare the quantification by flow cytometry and qRT-PCR. The results were very similar for both strains (S1 Fig). As qRT-PCR is a fast and reliable method, it was used for quantification of the viruses. One strain was chosen as prototype for each lineage, 287/11 for L1 lineage and 484/12 for L6 lineage.

**Fig 2:**
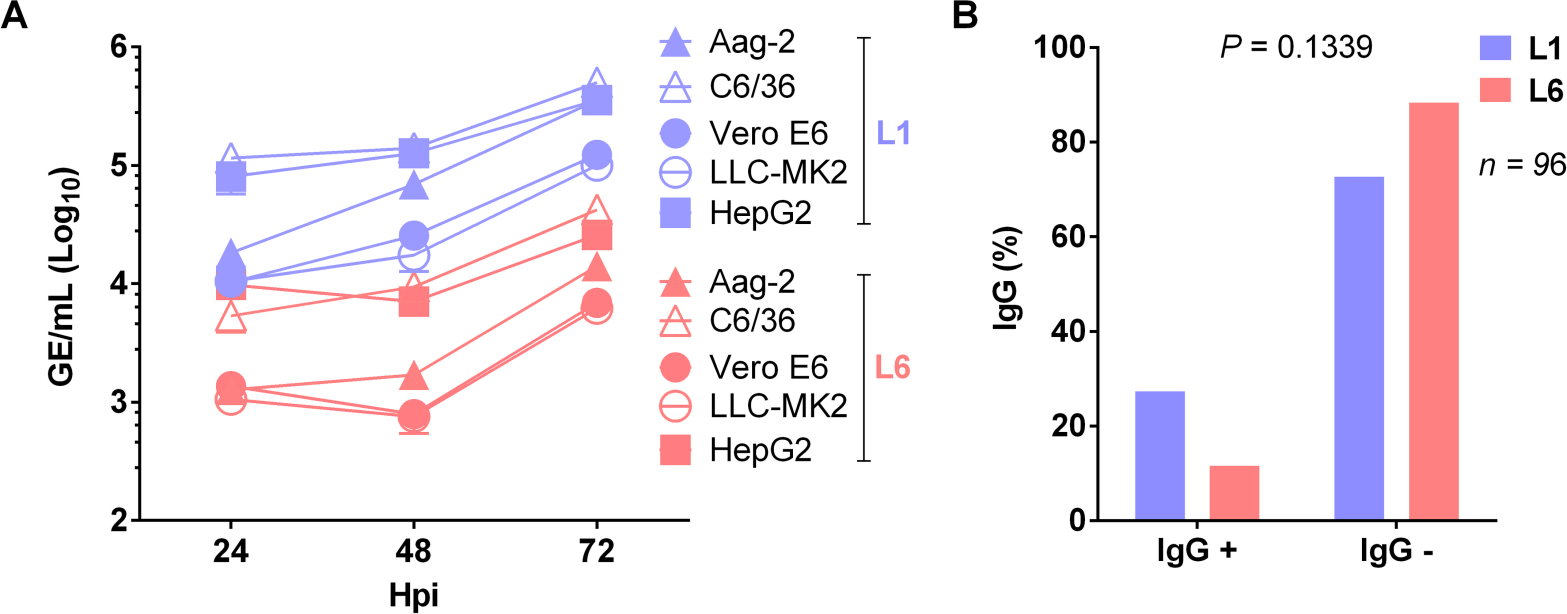
Replicative fitness in cell culture and cross-reactive immunity of DENV-1 lineages. (A) Growth curves of L1 or L6 isolates infected at an MOI of 0.1 in mosquito (C6/36 and Aag-2), human (HepG2) and non-human primate (Vero E6 and LLC-MK2) cell lines (*P* ≤ 0.05, Student’s T test). (B) Proportion of L1 or L6 DENV-1 cases classified as primary (negative IgG) or secondary (positive IgG) DENV infections (Fisher’s exact test).

In mosquito cell lines (C6/36 and Aag-2), L1 viruses had higher replication rates than L6 viruses by approximately one log_10_. Viral replication of L1 and L6 was also assayed in non-human primate cell lines (Vero E6 and LLC-MK2), in which similar results were obtained. Likewise, in a human cell line (HepG2), L1 viruses again demonstrated higher viral replication (Fig 2A).

The immunological status of the population could interfere with the propagation of viruses through an antibody-dependent enhancement (ADE) phenomenon [2]. Neutralizing antibody induced by previous heterologous infection can also interfere with DENV replication in vivo in an acute infection [21].

We tested 96 sera from symptomatic DENV-1 (L1 or L6)-infected patients for anti-dengue IgG antibodies to identify differences in the presence of prior heterologous exposure to DENV infection in both patient populations. Cross-reactive immunity can interfere with the risk of a second infection for a specific lineage of DENV-1, but primary (negative IgG) or secondary (positive IgG) DENV infections were similar in both groups (*P* = 0.1339; Fig 2B). Due to L6 persistence, we would have expected L6 viruses to replicate more efficiently (better replicative fitness) than L1 viruses. Therefore, an additional factor, such as transmission potential by the vector, could have been a contributor and were, therefore, investigated.

#### Replicative fitness in mosquitoes and coinfections

**Fig 3:**
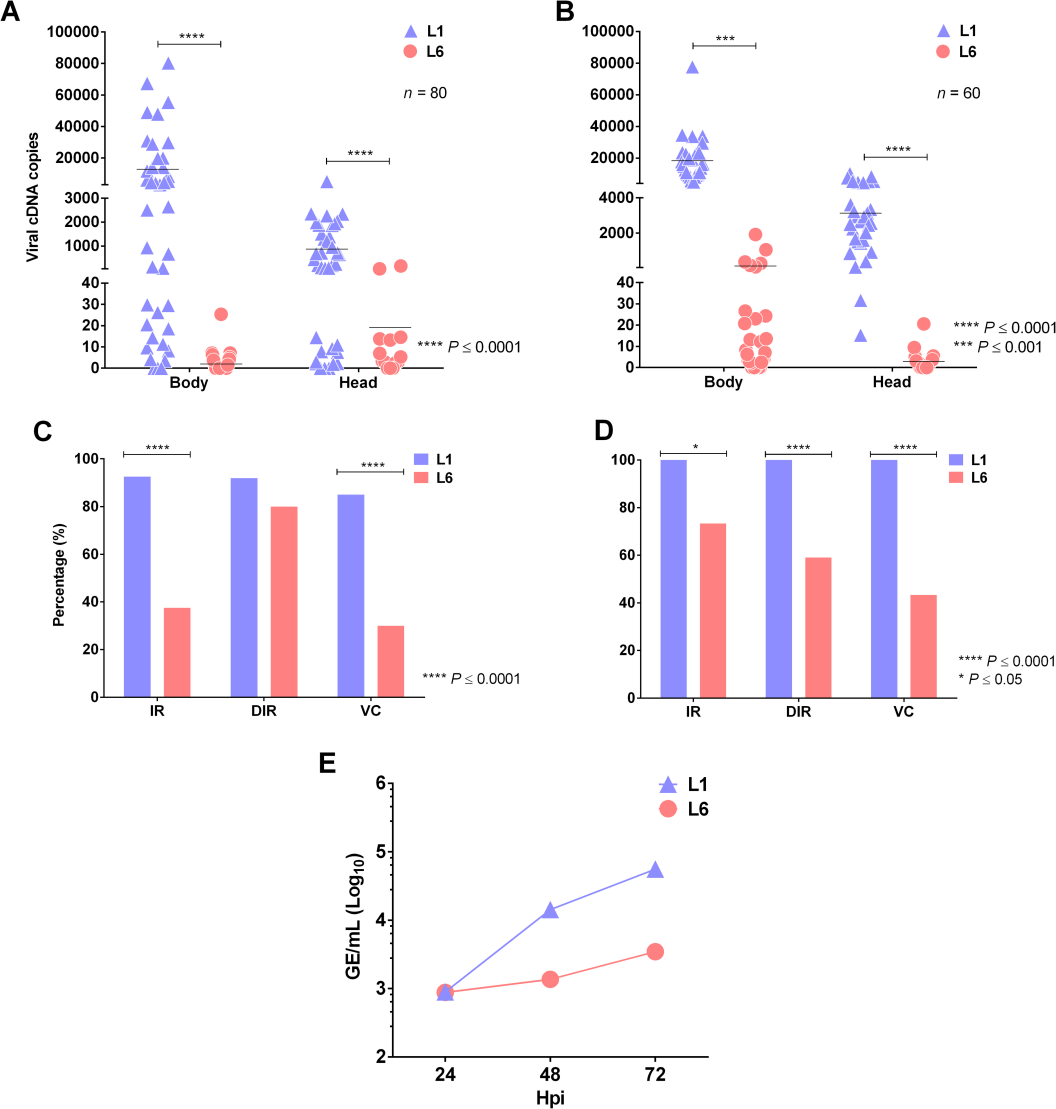
Replicative fitness in mosquitoes under selective pressure of DENV-1 lineages. (A and B) Viral cDNA copy number of L1 or L6 viruses in PPCampos. (A) and Dom Pedro (B). (C and D) Proportion of the infection rate (IR), disseminated infection rate (DIR) and vector competence (VC) of L1 or L6 viruses in PPCampos (C) and Dom Pedro (D). (E) Coinfection of L1 and L6 isolates in Aag-2 cells (MOI = 0.1) (Student’s T test).

For a virus lineage to establish a fitness advantage over others, it must be able to infect and disseminate in mosquitoes at a higher rate, thus establishing a higher transmission potential [16]. We infected *Ae*. *aegypti* mosquitoes from two populations: PPCampos (captive) and Dom Pedro (wild). Consistent with the results obtained in C6/36 and Aag-2 cells, we observed that both lineages infected the mosquitos, resulting in a greater number of genome copies of L1 viruses. Indeed, the levels of L1 viruses were greater than those of L6 viruses in either the bodies or the heads after single infections in PPCampos (Fig 3A) and Dom Pedro (Fig 3B) mosquitoes.

Next, we calculated the infection rate (IR), disseminated infection rate (DIR) and vector competence (VC) for each lineage. These calculations again demonstrated an advantage of L1 viruses (range: 85 to 100%; Fig 3C) over L6 viruses (range: 30 to 80%; Fig 3D).

After analyzing the viral replication of lineages in cell culture and mosquitoes, our results suggested that L1 displayed improved viral fitness, contradicting our epidemiological findings. However, caution should be exercised when interpreting *in vitro* experimental outcomes because they could fail to represent the complexity of natural transmission cycles, and differences in fitness can be demonstrated under forms of selective pressure such as coinfection [16]. To examine the potential of coinfection to explain our results, we performed a viral competition assay in which Aag-2 cells and mosquitoes were coinfected with L1 and L6 isolates and the relative amount of each virus was quantified. When present in culture concomitantly, L1 viruses replicated approximately one log faster than L6 viruses in Aag-2 cells, a statistically significant difference, except at 24 hours post infection (hpi) (*P* = 0.6081; Fig 3E and S4 Fig). These results suggest that viral-related factors or viral-specific fitness do not account for the dominance of the circulating L6 lineage without replacement by L1.

### Epidemiological fitness in viral dominance

#### Subgenomic flavivirus RNA and interferon response

DENV-2 lineages can inhibit type I interferon responses via subgenomic flavivirus RNA (sfRNA) production. In DENV-2, the greater expression of sfRNA relative to genomic RNA (gRNA) can be responsible for improved fitness [15]. Thus, higher sfRNA:gRNA production ratios could have accounted for the epidemiological fitness of DENV-1 in our population.

**Fig 4:**
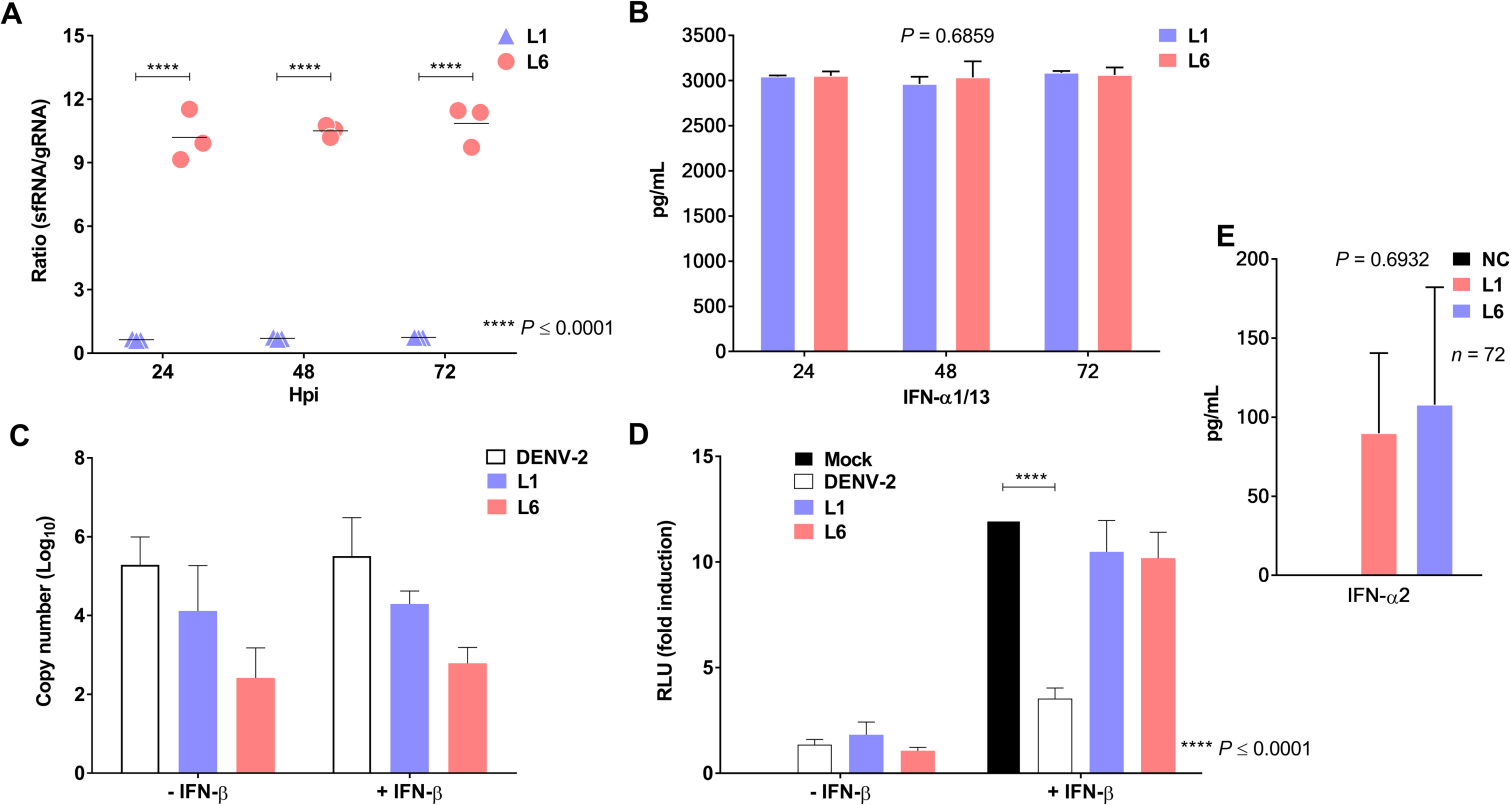
sfRNA and expression of type I interferon antiviral responses by DENV-1 lineages. (A) Ratio of sfRNA:gRNA in HepG2 cells infected with L1 or L6 viruses at an MOI of 1.0 (Student’s T test). (B) Quantification of IFN-α1/13 production in supernatants of HepG2 cells by an ELISA (Chi-squared test). (C) Viral cDNA copy number of DENV-2, L1 or L6 viruses in HBMECs (before or after treatment with IFN-β) using real-time PCR. (D) IFN-induced luciferase activity in HBMECs that were mock-treated or infected with DENV-2, L1 or L6 DENV-1 in the presence or absence of IFN-β (Student’s T test). (E) Quantification of IFN-α2 production in negative controls and L1 or L6 DENV-1-infected patients using the Luminex assay (Mann-Whitney test).

Evaluation of the sfRNA:gRNA ratio in HepG2 cells showed that the sfRNA:gRNA ratio in cells infected with L6 was 10-fold higher than that in cells infected with L1 (*P* ≤ 0.001; Fig 4A). The type I IFN antiviral response was measured to assess whether this mechanism could account for the epidemiological fitness of the DENV-1 lineages. In the supernatants of HepG2 cells, IFN-α1/13 levels were similar in both lineages (*P* = 0.6859; Fig 4B). To verify whether IFN signaling was impaired during infection, HBMEC-ISRE-Luc cells were infected with DENV-2 (strain 16681) and the L1 or L6 DENV-1 lineages, in the presence or absence of IFN-β (Fig 4C). The IFN response was reduced only in HBMEC-ISRE-Luc cells infected with DENV-2. Neither L1 nor L6 inhibited the IFN-induced response upon endothelial cell infection (Fig 4D). IFN-α2 was also measured in naturally infected human sera and, consistent with the results obtained in cell culture, there were no differences in IFN-α2 production between patients infected with either L1 or L6 (*P* = 0.6932; Fig 4E). According to our findings, both lineages were unable to inhibit IFN expression or the IFN-induced response. Thus, the proposed model for epidemiological fitness based on sfRNA [15] does not apply to these DENV-1 lineages.

#### Cytokine profiles

Because viral fitness and suppression of the type I IFN response could not account for our epidemiological findings, we focused on evaluating the immune responses in patients infected with either lineage of DENV-1. The most immunogenic lineage would be replaced in a given population because enhanced immune responses would result in decreased viral loads and a lower chance of transmission in humans. Initially, sera from 72 patients presenting with dengue without warning signs, infected with either L1 or L6 lineages, were tested for 29 cytokines, chemokines, adhesion molecules and growth factors.

**Fig 5:**
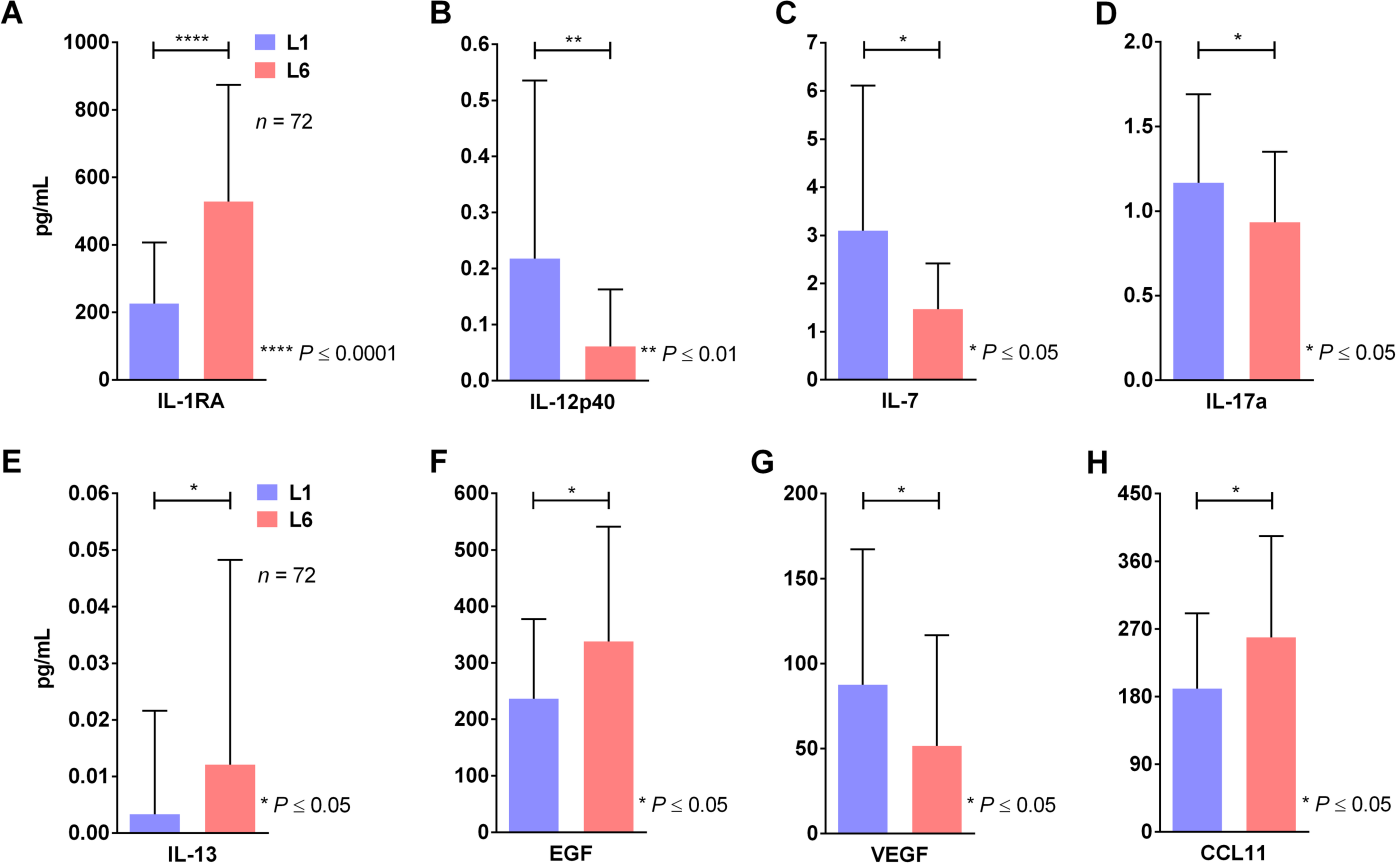
Quantification of cytokines, chemokines, adhesion molecules and growth factors showing significant differences in L1 or L6 DENV-1-infected patients. (A) IL-1RA. (B) IL-12p40. (C) IL-7. (D) IL-17a. (E) IL-13. (F) EGF. (G) VEGF. (H) CCL11. (Mann-Whitney test). See also S3 Fig.

The levels of various molecules were detectable in the serum samples, but only the levels of cytokines IL-1RA, IL-12p40, IL-7, IL-17a, IL-13, EGF, VEGF and CCL11 (Fig 5A–5G) showed differences between the serum of patients infected with the L1 lineage and those infected with the L6 lineage. Whereas the levels of IL-12p40, IL-7, IL-17a and VEGF were elevated in patients infected with L1, the levels of IL-1RA, IL-13, EGF and CCL11 were greater in patients infected with L6. None of the other tested molecules showed significant differences (S3 Fig).

#### Antigenicity and adaptive immune response

**Fig 6:**
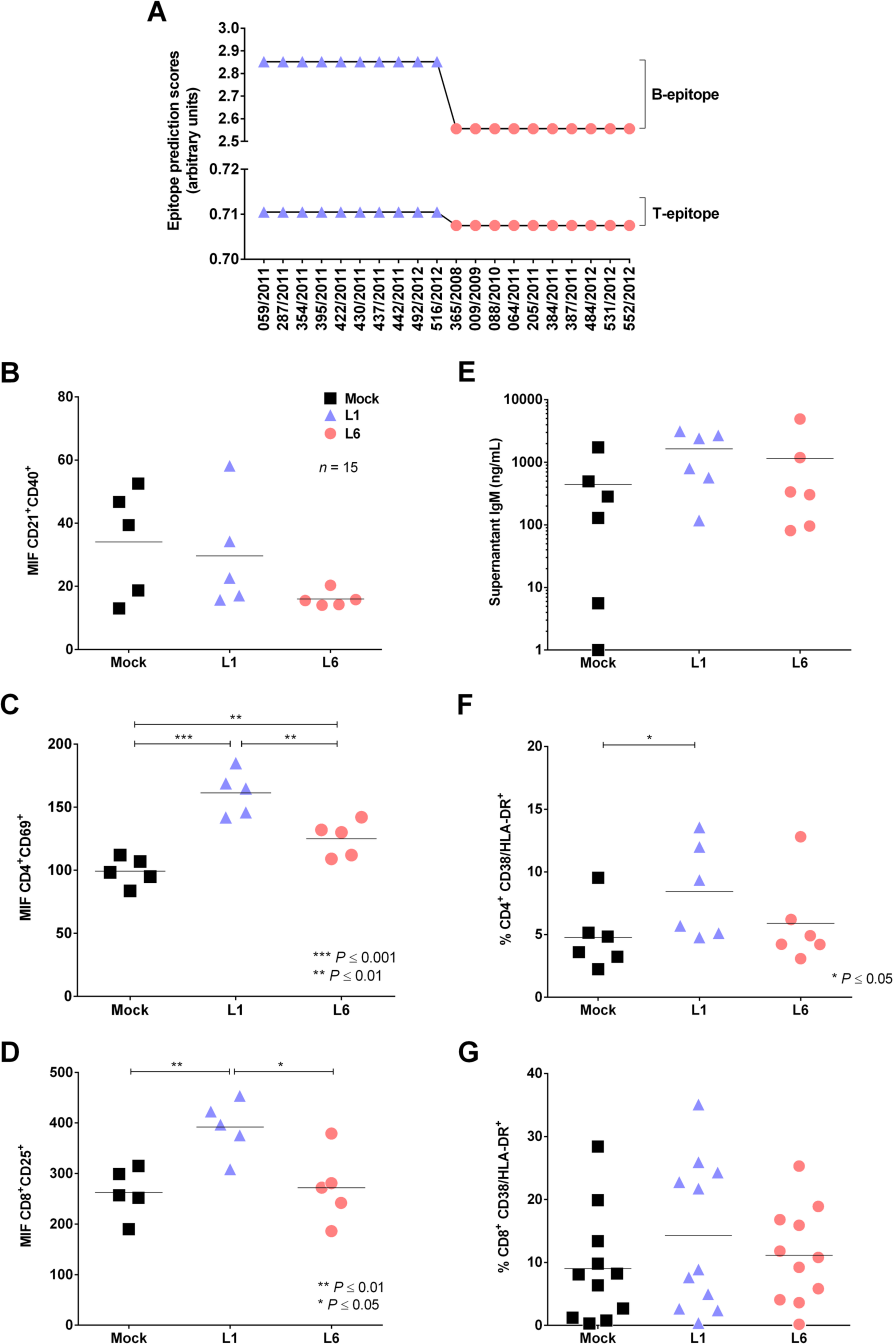
Rank of antigenicity, B and T cell activation and viral load of DENV-1 lineages. (A) Twenty DENV-1 sequences from SJRP (L1 and L6) subjected to B and T antigenicity prediction and classified according to their immunogenic potential. (B, C and D) Analysis of B (B), CD4 (C) and CD8 T (D) cell activation in C57BL/6 at 7 days post-infection by flow cytometry (Student’s T test). (E, F and G) Average and individual data for IgM levels by an ELISA (E); CD4 (F) and CD8 T (G) cell activation frequency obtained in PBMCs from healthy donors by flow cytometry (Student’s T test).

Previous studies [22] hypothesized that lineages with lower antigenic potential could have better epidemiological fitness. Thus, we conducted an *in silico* analysis using 20 DENV-1 sequences that had been previously clustered into the L1 and L6 lineages to verify potential antigenic differences in polyprotein. We found that four aa substitutions in the sequences grouped in the L1 lineage increased the B and T epitope antigenic scores. Sequences clustered into L6 displayed diminished antigenicity (Fig 6A); therefore, L1 would trigger the immune system in a more effective manner.

To confirm *in vivo* the reduced antigenicity observed for the L6 sequences using bioinformatics, eight-week-old male C57BL/6 mice were immunized with L1 and L6 isolates, and spleen cells were collected to analyze B and T cell activation by flow cytometry. B cell activation was measured by analyzing CD21^+^/CD40^+^ expression, and L6 did not appear to activate B cells (Fig 6B). An analysis of CD4 and CD8 expression demonstrated that L1 induced a much higher frequency of activated CD4^+^/CD69^+^ (Fig 6C) and CD8^+^/CD25^+^ cells (Fig 6D) than L6, indicating prominent T cell activation.

To confirm whether the L1 and L6 isolates would differentially stimulate human leukocytes, PBMCs from 11 healthy donors were mock-treated or infected with each isolate and B and T lymphocyte activation was analyzed; anti-DENV serology was performed in 9 donors to determine whether they were dengue naive or had been previously infected; serum was not available for 2 donors. Among those 9, seven were dengue naive, one showed anti-DENV IgG (but we could not evaluate the serotype nor whether there was primary or secondary infection), and one showed inconclusive serology result.

B cell activation was evaluated by measuring total IgM levels in culture supernatants. We observed that L1 induced slightly higher, but not significant levels of IgM, in comparison to L6 (Fig 6E). Regarding CD38 and HLA-DR expression on T cells, we observed that 8 donors showed a higher T cell activation in response to L1 than to L6 isolates; those included six dengue naive and two with undetermined serology. Four donors showed a slightly higher response induced by L6; those included two dengue naive, one dengue positive, and one inconclusive (Fig 6F and 6G).

We also measured the levels of IFN-γ, IL-6 and IL-8 in PMBC culture. Analysis of the cytokines secreted demonstrated that both lineages induced high levels of IFN-γ and IL-6; but higher levels were detected in L1-infected cells. On the other hand, L6-infected cells showed increased IL-8 (S2 Fig).

**Fig 7:**
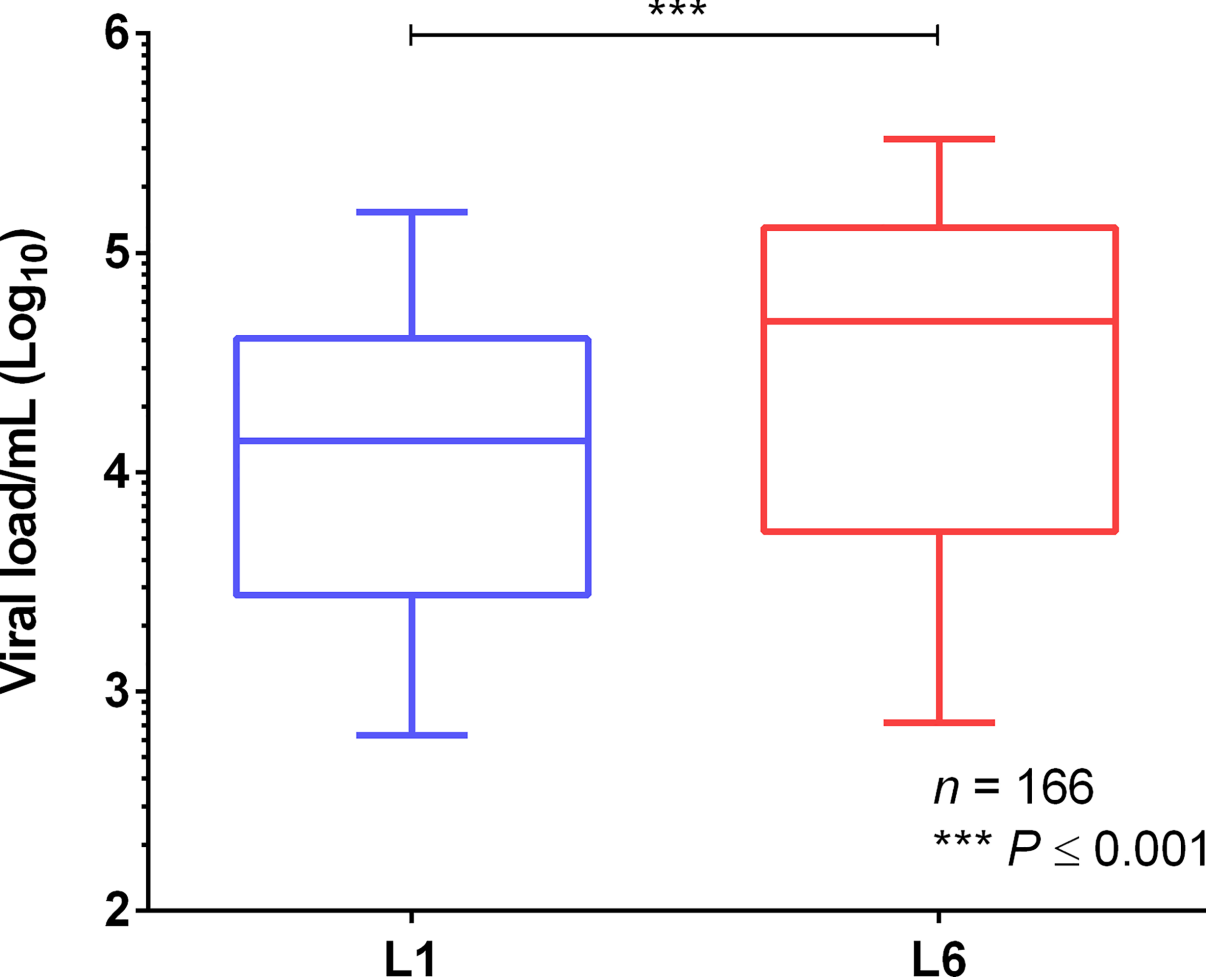
Viral load quantification of DENV-1-infected patients using real-time PCR.

Because effective innate and adaptive immune responses would eventually translate into the control of viral replication, we evaluated the levels of virus in serum samples of patients infected with the L1 or L6 DENV-1 lineages from 2008 to 2014. As shown in Fig 7, the levels of virus in patients infected with L6 were 3.5-fold higher in serum than in patients infected with L1 viruses (*P* ≤ 0.001).

## Discussion

To understand the forces that drive DENV epidemics, it is crucial to develop efficient control methods for this disease. The introduction of new lineages and CR is important for maintenance of the disease in a given population. CR is a well-documented event that occurs in many epidemics when a new DENV strain emerges and displaces the endemic strain, even between viruses of the same serotypes and genotypes. This process usually causes an increase in both the number of cases and the severity of the disease [15,16]. Although relatively common, the forces that dictate whether CR will occur are not completely understood. Genetic variations may alter viral fitness; however, the interplay of this factor with epidemiological factors has not been defined.

Since 2008, the L6 lineage of DENV-1 (genotype V) has circulated in SJRP. The duration of the circulation of this lineage in the population allowed us to consider it an endemic lineage. In mid-2010, a different Brazilian lineage arose: L1. This newly introduced lineage appeared to have improved viral fitness compared to L6; thus, L1 was expected to replace L6 as the dominant strain. From the emergence of the L1 lineage until 2012, the epidemics in SJRP appeared to follow the path of other epidemics, and the CR event would occur. However, the L1 lineage did not displace L6, but rather both co-circulated without a clear predominance. L1 started to decline until its complete disappearance in 2013. This finding raised questions about differences in the fitness between the lineages, which were investigated by our group.

Lineages of the same serotype may present different characteristics in transmissibility, virulence and antigenic properties, some of which may lead to increased fitness and could be related to a greater epidemic potential [15,16,23]. The viral fitness is defined by *the capacity of a virus to produce infectious progeny in a given environment*, *whereas the capacity of a virus to become dominant in a given region has been called epidemiological fitness* [15]. The latter is determined by a combination of factors, including the genetics of the viral strain, transmission potential by the vector and human system interactions [24]. Investigations of the factors involved in lineage replacement have revealed important implications for advancing our understanding of DENV epidemiology, evolutionary dynamics and control [11].

Viral fitness is an important contributing factor to CR and, on some occasions, it can account for the persistence of the strain. In other DENV epidemics, improved replicative fitness of the emerging strain has been responsible for the dominance of the newer lineage. For example, the replicative fitness advantage of the NI-2B lineage over the NI-1 lineage in *Ae*. *aegypti* could have contributed to the CR event, resulting in the dominance of NI-2B in Managua [16]. Replacement of the NI-1 lineage by the NI-2B lineage was also associated with an increase in disease severity.

In the SJRP epidemic, differences in the replicative fitness could have accounted for the persistence of L6 even after the introduction of the new lineage (L1). However, the L1 had higher replication rates than L6 in mosquito, human and non-human primate cell lines. It also had an increased fitness in two different populations of *Ae*. *aegypti* mosquitoes and thus could have a higher potential for transmission than L6. Unfortunately, it was not possible to analyze viral levels in mosquitos collected at that time. In theory, the coinfection of vectors with both lineages can result in competition and can accurately represent the natural transmission cycles. However, the superior viral fitness of L1 in mosquito cell culture indicates that coinfections do not have any effect on viral fitness. In fact, all tests conducted by our group revealed superior viral fitness of L1, *in vitro* or *in vivo*. Therefore, the better replicative fitness L1 would induce its dominance and not its replacement by L6. Some studies theorize that *in vitro* infections can sometimes fail to predict the actual dynamics of DENV epidemics [16]. Therefore, differences in replicative fitness did not explain the observed pattern of L6 persistence.

Evasion of interferon responses by the virus could be a factor underlying the maintenance of the L6 lineage in the population. This has been identified as a determinant of epidemiological fitness in the lineage dominance of DENV-2 (PR-2B) in Puerto Rico [15]. During flavivirus infections, short flavivirus RNAs (sfRNAs) are produced by the incomplete degradation of viral RNA by the host-cell exonuclease Xrn1. These sfRNAs are associated with the pathogenesis of flaviviruses [25]. The high expression of sfRNAs could have inhibited type I interferon responses and facilitated evasion of the immune response. In a study conducted in Puerto Rico, the PR-2B lineage produced elevated expression of subgenomic flavivirus RNA (sfRNA) relative to genomic RNA (gRNA) during replication. Notably, sfRNA could bind TRIM25 and inhibit the IFN response. Thus, higher sfRNA:gRNA ratios are associated with superior epidemiological fitness [15]. Although L6 had higher sfRNA:gRNA ratios than L1, which in turn could increase its epidemic potential, it did not appear to be related to IFN inhibition, as previously reported [15]. Both lineages appeared to be incapable of inhibiting the IFN response and produced similar levels of type I IFN *in vitro* and *in vivo*, suggesting this was not the mechanism underlying the differences observed.

Previous antibody immunity to dengue could also play a role in epidemic dynamics. For example, the immunological status of the population could interfere with L1 propagation through an antibody-dependent enhancement (ADE) phenomenon. Waning immunity due to a prior DENV infection can alter the outcome of the present infection, as observed in Nicaragua [2]. High titers of pre-infection cross-reactive neutralizing antibodies drastically reduce the probability of a severe disease in a second infection, although this protective effect is not observed against all DENV serotypes [26]. However, no significant differences in the frequency of anti-dengue IgG antibodies were found in our patients.

Because previously reported mechanisms were unable to explain the persistence of L6, we evaluated various aspects of the immune response in order to provide an alternative explanation to our findings. To assess possible differences in immunological responses, a panel of 29 cytokines, chemokines, adhesion molecules and growth factors were measured in plasma of patients. Dengue infection was associated with the increase of several cytokines in serum of patients. Overall infection with the L6 lineage was associated with greater increases of cytokines with anti-inflammatory (IL-1RA and IL-13) or Th2-like activity (IL-13 and CCL11). In contrast, infection with the L1 lineage was associated with greater increase of cytokines with Th1/Th17–like activity (IL-12 and IL-17) and IL-7, a cytokine that promotes lymphocyte development in the thymus and maintains survival of naive and memory T cell homeostasis in the periphery. The exact role of these cytokines in the context of dengue infection is not precisely know but these results clearly show that the L1 lineage had a tendency to generate immune responses usually associated with resistance to dengue infection whereas the L6 lineage the tendency to generating cytokines with anti-inflammatory activity or that block Th1/Th17 responses.

The latter studies suggested that immune responses to L1 were enhanced and more pro-inflammatory and led us to investigate in greater detail adaptive immune responses in patients infected with the L1 or L6 lineages. *In silico* analysis suggested that the L1 lineage was potentially more immunogenic than the L6 lineage. These predictions were confirmed by studies in mice that showed increased immunogenicity of L1, as assessed by greater B and T cell activation. Unfortunately, we did not have access to PBMCs derived from L1 or L6 infected patients, and only the sera were available. Therefore, it was not possible to assess directly T cell recall responses to L1 and L6 viruses. However, experiments in human PMBCs infected with either lineage showed that the L1 lineage induced stronger responses than those infected with L6 viruses. Altogether, he data suggest that different DENV isolates might induce distinct levels of B and T cell activation. L1-induced response was mostly associated to IFN-γ production, whereas L6 induced activation was driven to inflammatory IL-8 secretion. Distinct T cell phenotype and function, with increased T cells activation and IFN-γ production might then be associated to disease control [27–30]. Altogether, these studies suggest that the adaptive immune responses in infected individuals triggered by L1 were stronger than those triggered by L6.

Our prediction would be that differences in immune response would necessarily have to associate with altered viral loads, if these responses were to affect the likelihood of one strain to override the other. Indeed, patients infected with the L6 lineage, the one with significantly decreased immune activation, were those with the higher viral loads. We would expect that the high viral L6 loads would enhance the probability of transmission of the L6 lineage to the vector and, consequently, to another host.

The current dogma indicates that, after infection with a certain DENV serotype, only a heterologous DENV serotype can cause infection in the same individuals. However, studies using non-human primates have indicated that new inoculations with either the same or different genotypes of DENV-2 can cause a persistent boost in neutralizing antibodies [31]. Because L6 only weakly stimulates B and T cells, it may not increase immunological memory, nor may it induce the development of neutralizing antibodies in sufficient titers to protect against a new exposure. Although further work with specific experiments are needed to strengthen this evidence, it appears that neutralizing antibodies may quickly control infections, preventing more severe disease; however, they may not avoid future infections. A similar mechanism has been proposed for studies in Nicaragua [31] and may provide an explanation for the maintenance of L6 for such a long period in the population. High viral loads of L6 enhance the probability of vector transmission of the L6 lineage. However, despite the better replicative fitness, the L1 lineage appears to elicit a stronger immune response, preventing broader propagation of this lineage due to the extinction of the susceptible population.

**Fig 8:**
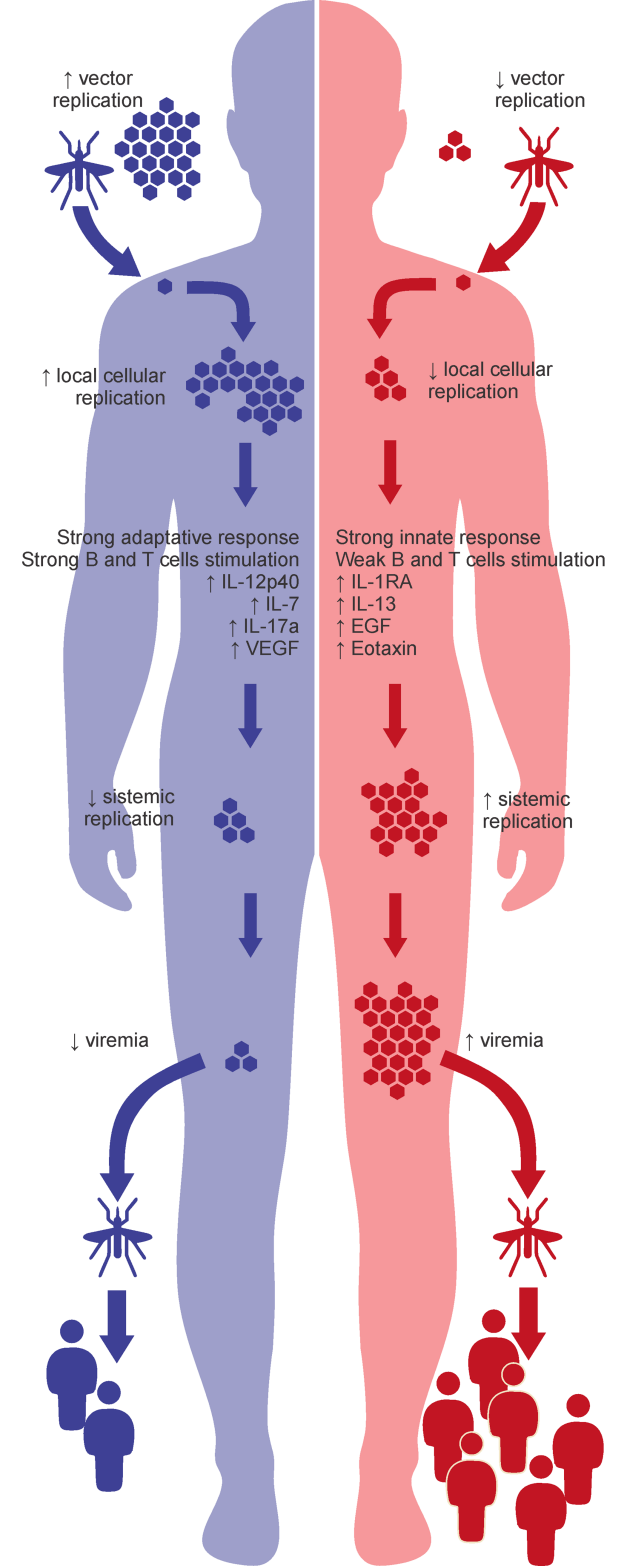
Proposed model for L6 lineage (red) dominant circulation without L1 lineage replacement (blue) in the SJRP population, with the main differences found between the two DENV-1 lineages.

Based on our data, the absence of CR together with the superior epidemiological fitness of L6 in SJRP was a result of the human immune system functioning as a bottleneck that favored the L6 lineage to achieve a broader distribution, even with lower viral fitness. The model is summarized in Fig 8. The inability of the L1 lineage to replace the endemic L6 lineage in this city shows that the interplay between replicative, immunological and epidemiological features that affect the dynamics of viral propagation is far more complex than previously suspected. In this case, the lower viral immunogenicity for B and T cells associated with host immunological factors counteracts the superior viral fitness, contributing to the lineage dominance.

## Materials and methods

### Ethics statement

The panel of 186 DENV-1 viruses used in the present study was collected from symptomatic patients from 2008 to 2015 in a public healthcare facility in SJRP (São Paulo, Brazil) as part of the flavivirus and hantavirus surveillance program in the city. This study was reviewed and approved by the Human Research Ethics Committee of Faculdade de Medicina de São José do Rio Preto (CAAE: 02078812.8.00005414).

All the samples are obtained from an existing collection in the laboratory (LPV-Dengue 2008–2015) and all of them were already anonymized.

Blood samples (buffy coats) from healthy donors were obtained anonymously from the Hemotherapy Service at the Hospital Universitário Clementino Fraga Filho (HUCFF) of Universidade Federal do Rio de Janeiro (UFRJ). The study protocol was approved by the Experimental Ethics Committee of UFRJ (Permit Number: IMPPG 025), and the review board waived the need for informed patient consent.

All animal work was performed in accordance with the Fiocruz Animal Use Committee (protocol P-60/14-4; license number LW-30/15). Fiocruz personnel are required to adhere to applicable federal, state, local and institutional laws and policies governing animal research, including the regulations from the Brazilian Council of Animal Use Control (CONCEA - 3rd Edition, published at Sep, 26, 2016), Federal Law 11794/08 and Protocols for Animal Use—Oswaldo Cruz Research Foundation (ISBN: 85-7541-015-6).

### Samples

We observed predominantly dengue without warning signals. All the samples used in this study were collected untill 5 days of the *symptoms* onset. The serum samples were subjected to molecular biological and serological diagnostic tests for dengue. Initially, DENV infection was confirmed using reverse transcription-PCR (RT-PCR) and multiplex nested PCR (M-N-PCR) assays as described previously [32], which distinguishes the four serotypes. Ninety-six DENV-1-positive samples were tested for anti-dengue IgG antibodies, as recommended by the SERION ELISA classic Dengue IgG test kit (Virion Serion).

### DENV envelope gene sequence amplification and analysis

Twenty DENV-1-positive samples were used to amplify the envelope gene sequence, followed by nucleotide sequencing using the Sanger-based method with a previously described primer [33]. Viral RNA was extracted using the QIAamp Viral RNA Mini kit (Qiagen) as recommended by the manufacturer. First-strand cDNA was synthesized using the Superscript III First Strand Synthesis System (Invitrogen) following the manufacturer’s instructions with primer d1a16. PCR was performed to amplify a 1,855-bp fragment, of which 1,485 bp corresponded to the entire DENV-1 E gene. The reaction consisted of 2 μL of cDNA, 5 μL of 10X Accutaq LA buffer, 2.5 μL of dNTPs (10 mM/μL), 1 μL of DMSO 2%, 1 μL of primers d1s3 and d1a17 (10 μM), 0.5 μL of Accutaq LA DNA polymerase (5 U/μL; Sigma-Aldrich) and DEPC-treated water. The reactions were submitted to the following cycle conditions: 98°C for 30 sec, followed by 30 cycles of 94°C for 15 sec, 50°C for 20 sec and 68°C for 1 min and 30 sec. A final extension step was performed at 68°C for 10 min.

An analysis of the amplicons was performed by electrophoresis on a 1% agarose gel. The PCR product (40 μL) was purified using 2.8 μL of 3 M sodium acetate and 1.2 μL of cold absolute ethanol. The samples were stored overnight at -20°C or for 1 h at -80°C and subsequently centrifuged at 16,100 x g for 20 min. The pellet was washed with 200 μL of 70% ethanol and centrifuged at 16,100 x g for 10 min. The dried pellet was resuspended in 20 μL of Milli-Q water. Twenty nanograms of purified PCR products were used as templates in 20 μL of cycle sequencing reactions using 2 μL of 1X Sequencing buffer, 2 μL of BigDye Terminator v.3.0 (Applied Biosystem), 1 μL of forward and reverse primers (3.2 μM) (see S1 Table) and DEPC-treated water; these samples were submitted to 96°C for 1 min followed by 25 cycles of 96°C for 10 sec, 50°C for 5 sec and 60°C for 4 min. Precipitation of sequencing reactions was performed using ethanol/EDTA as recommended by the BigDye Terminator kit v.3.0 (Applied Biosystems) protocol. The samples were resuspended in 10 μL of Hi-Di Formamide (Applied Biosystems) and analyzed with ABI PRISM 3130 equipment (Applied Biosystems).

The quality of the sequences was analyzed using Sequencing Analysis 5.2 software (Applied Biosystems). The consensus sequence was edited using Accelrys Gene v.2.5 (Accelrys). Nucleotide sequences were then aligned with the previously published E gene sequence from GenBank (GU131863.1) using MEGA 6.0.6 Molecular Evolutionary Genetics Analysis (http://en.bio-soft.net/tree/MEGA.html).

To obtain insight into the genetic relationship among DENV-1 strains, envelope sequences obtained from different DENV-1 isolates from SJRP and other geographic sites were aligned using ClustalW [34], taking into account the codon sequences. Amino acid sequences were predicted, and substitution patterns were analyzed. Phylogenetic and coalescent analyses were conducted using BEAST package v.1.8 with Markov Chain Monte Carlo (MCMC) algorithms [35]. Input files for BEAST were generated using BEAUTI v.1.8.1 [35], and the year each strain was isolated/obtained was used as a calibration point. Analyses were performed using the General Time Reversible nucleotide substitution model with four gamma categories (GTR + 4G), the Bayesian Skyline method [36] and a relaxed (uncorrelated lognormal) molecular clock. Two independent runs (100 million chains, discarding the first 10 million steps) were run, and parameters and trees were sampled every 10,000 steps. The convergence of parameters was checked with Tracer v1.6.0 [37], and uncertainties were addressed as 95% Bayesian Credible Intervals (BCI). Using Tree Annotator v. 1.8.1 [38], a maximum clade credibility (MCC) tree was generated and then visualized in Figure Tree v. 1.4.2 [38].

In addition, full-length sequences of viral RNA genomes from SJRP were sequenced using next-generation sequencing with Illumina **MiSeq System (Illumina)** as described previously [39]. Briefly, isolates were subjected to RNA extraction using the QIAamp Viral RNA Mini kit (Qiagen), followed by quantification using a PicoDrop (Picodrop Limited). RNA was treated with DNAse I (Sigma-Aldrich), and reverse transcription-PCR was performed with random primers (50 ng; Invitrogen) using the High Capacity cDNA Reverse Transcription kit (Applied Biosystems), according to the manufacturer's instructions. After sequencing, the obtained sequences were assembled and edited using Geneious v.7.1.4 (Biomatters Ltda), and polyprotein sequences were aligned and translated to compare aa substitutions. Two sequences (287/2011 and 484/2012) were selected to represent the L1 and L6 lineages, respectively.

### Genotyping

When it was not possible to obtain an amplicon of the envelope gene region or complete genomic sequences, lineage discrimination of DENV-1-positive samples was performed using the TaqMan Real-Time PCR genotyping assay and the AgPath-ID One-Step RT-PCR kit (Applied Biosystems) with two primers and probe sets for the envelope (2021_F, 2021_R and 2021_P1) and NS5 (8587_F, 8587_R and 8587_P2) regions, as shown in S2 and S3 Tables.

For the genotyping assay validation, twenty samples previous grouped into L1 or L6 lineages by phylogenetic analyses were selected and the experimental validation was performed under blind conditions. All of the samples used were correctly discriminate.

The One-Step qRT-PCR reaction was performed using two different master mixes and consisted of 7 μL of RNA sample, 12.5 μL of 2X RT-PCR buffer, 1 μL of forward and reverse primers (20 μM), 0.75 μL of probe (10 μM), 1 μL of 25X RT-PCR enzyme mix and nuclease-free water in a total volume of 25 μL per reaction. To identify the L1 lineage, primers 2021_F and 2021_R and probe 2021_P1 were used; 8587_F, 8587_R and 8587_P2 were used for lineage L6 identification. The reactions were subjected to the following cycle conditions, with data collections at 60°C: 50°C for 10 min, 95°C for 10 min, 60°C for 30 sec and 95°C for 10 min, followed by 50 cycles of 95°C for 15 sec and 60°C for 1 min. A final extension step was conducted at 60°C for 30 sec. Genotyping was performed using human sera and standardized samples (to provide standard curves) to allow the relative quantification of virus levels. All qRT-PCR reactions were performed using a StepOne Real-Time PCR System (Applied Biosystems).

### Cells and viruses

Peripheral blood mononuclear cells (PBMCs) were obtained after centrifugation of buffy coat samples over a ficoll-hypaque gradient. Moreover, human brain microvascular endothelial cells (HBMECs) were stably transfected with the reporter vector pISRE-Luc-Hygro containing an NdeI-Bst1107 site of pISRE-Luc (Stratagene) and cloned into vector pCEP4 (Invitrogen). Cells then referred to as HBMEC-ISRE-Luc were kindly provided by Dr. Laura Helena Vega Gonzales Gil, CPqAM, FIOCRUZ, Recife-PE, Brazil. Both human primary cells were cultured in RPMI-1640 medium (Cultilab) supplemented with 10% fetal calf serum (FCS; Gibco) (complete medium) at 37°C in a 5% CO_2_ atmosphere.

Mosquito, human and non-human primate cell lines were also used for the *in vitro* assays. C6/36 cells (ATCC) were cultured in Leibovitz's medium (L-15; Cultilab) and Aag-2 (kindly provided by Dr. João Trindade Marques, UFMG, Brazil) in Schneider’s insect medium (Sigma-Aldrich) at 28°C. Vero E6 and LLC-MK2 cells (ATCC) were cultured in Minimum Essential Medium (MEM; Cultilab) and HepG2 cells (ATCC) in Dulbecco's Modified Eagle's Medium (DMEM; Cultilab) at 37°C in a 5% CO_2_ atmosphere. All culture mediums were supplemented with 1% fetal bovine serum (FBS; Gibco) for maintenance or 10% for expansion, excluding Aag-2, for which 8% FBS, 10 U/mL of penicillin, 10 g/mL of streptomycin and 250 μg/mL of amphotericin B were used (Gibco).

Initially, samples were selected to represent each lineage, and viral isolation was performed based on previous investigations [40]. Briefly, viruses selected from L1 or L6 DENV-1 human sera were diluted 1:10 in L-15 and used to inoculate C6/36 cells, which were then incubated at 28°C for 7–10 days. Successful isolation was confirmed by RT-PCR of the culture supernatant, as previously described for the sequencing reaction, followed by PCR. PCR was performed to amplify an 1,855-bp fragment using 2 μL of cDNA, 5 μL of 10X buffer, 2 μL of dNTPs (10 mM/μL), 2 μL of primers d1s3 and d1a17 (10 μM), 1 μL MgCl_2_, 0.25 of Taq DNA polymerase (5 U/μL; Sigma-Aldrich) and DEPC-treated water. The reactions were subjected to the following cycle conditions: 94°C for 2 min, followed by 30 cycles of 94°C for 45 sec, 56°C for 45 sec and 72°C for 45 sec. A final extension step was performed at 72°C for 10 min. Amplification was confirmed by electrophoresis on a 1.5% agarose gel.

Titration was determined by flow cytometry to calculate the number of infectious particles/mL (IP/mL), as described previously [41] with modifications, using FACSCalibur (BD Biosciences) equipment. The adaptations were cells fixed in 4% paraformaldehyde and permeabilized with 0.1% triton X-100. Viral stocks from the third passage were used for the experiments.

### Growth curves

Approximately 0.05x10^6^ cells (C6/36, Aag-2, Vero E6, LLC-MK2 and HepG2) were plated in each well of a 24-well plate 24 h prior to infection. Five cell lines were infected with L1 or L6 isolates at a multiplicity of infection (MOI) of 0.1 for 1:30 h in triplicates. The cells were then washed with 1X phosphate-buffered saline (PBS) to remove unabsorbed virus and then incubated in 1 mL of maintenance medium. The supernatants were harvested at 24, 48 and 72 hpi for relative quantification using the SYBR Green Real-Time PCR assay with the GoTaq qPCR Master Mix kit (Promega) and primers Den_F (5’-TTAGAGGAGACCCCTCCC-3’) and Den_R2 (5’-GAGACAGCAGGATCTCTGG-3’), as previously described [42]. Total RNA was extracted using TRIzol (Invitrogen) according to the manufacturer’s protocol. First-strand cDNA was synthesized using the Superscript III First Strand Synthesis System (Invitrogen) following the manufacturer’s instructions with primer Den_R2. The qRT-PCR reaction was also performed according to the manufacturer’s protocol.

The results were obtained using a standard curve and analyzing the melting curve (~ 85°C) to approximately CT 35, according to the minimum information for the publication of quantitative real-time PCR experiments (MIQE).

### Mosquito infection

*Ae*. *aegypti* eggs from populations PPCampos (captive; maintained approximately 15 years in an insectary at the Laboratório de Entomologia Médica, CPqRR, FIOCRUZ, Belo Horizonte-MG, from Campos dos Goytacazes-RJ) and Dom Pedro (wild; collected in 2014 in district Dom Pedro of Manaus-AM) were used in this study according to a previous description [43]. Briefly, the larvae were hatched in an insectary at a temperature of 28°C and relative humidity of 80%, and infections were performed using 3 to 5-day-old female mosquitoes (Dom Pedro from the F2 generation) using a glass feeding device containing 2/3 of blood mouse (*Mus musculus*) and 1/3 of C6/36 cells suspension infected with either L1 or L6 lineages.

The mean viral titer used for infection with L1 or L6 isolates was 5×10^5^ TCID_50_/mL. Infected PPCampos (n = 80) and Dom Pedro (n = 60) females were maintained in cages with 10% glucose solution until day 14 after the experimental infection (complete extrinsic incubation period). They were then dissected, and total RNA was extracted from their bodies and heads (with attached salivary glands) using TRIzol (Invitrogen) as described previously [40], followed by one-step qRT-PCR [43].

The infection rate (IR) was then calculated as the individual proportion of all experimentally infected mosquitoes, in which DENV was detected in the body. Similarly, the vector competence (VC) was calculated, in which DENV was detected in the head (indicating the virus escaped the midgut barrier, completing its life cycle). However, the disseminated infection rate (DIR) is the proportion of DENV-infected mosquito heads of all infected mosquitoes with virus dispersed in the body (DIR = VC/IR).

### Viral competition assays

Aag-2 cells were coinfected with L1 and L6 isolates mixed at an equal ratio (1:1) at an MOI of 0.1, and the supernatants were harvested at 24, 48 and 72 hpi, as previously described for the growth curves. Total RNA was extracted using TRIzol (Invitrogen) according to the manufacturer’s instructions, and the relative amounts of L1 and L6 viruses in each dual infection were calculated based on the developed genotyping assay.

### Investigation of sfRNA:gRNA ratios and IFN responses

HepG2 cells were infected at an MOI of 1.0, and the supernatants and cells were harvested at 24, 48 and 72 hpi. The sfRNA:gRNA ratio of the cells was obtained by real-time PCR using GoTaq qPCR Master Mix (Promega) with primers D1GSF, D1SF and D1GSR (described in S4 Table).

Cells total RNA was extracted using TRIzol (Invitrogen) according to the manufacturer’s protocol. First-strand cDNA was synthesized using M-MLV Reverse Transcriptase (Invitrogen) following the manufacturer’s instructions with primers D1GSR. The qRT-PCR reaction was performed using two different mixes, and it consisted of 5 μL of cDNA, 12.5 μL of 2X GoTaq qPCR Master Mix, 2 μL of forward and reverse primers (10 μM), 0.25 μL of CXR reference dye and nuclease-free water to a final volume of 25 μL per reaction. To quantify gRNA, primers D1GSF and D1GSR were used; otherwise, D1SF and D1GSR were used for sfRNA quantification. The reactions were subjected to the following cycle conditions, with data collected at 55°C and 60°C: 95°C for 5 min, followed by 40 cycles of 95°C for 15 sec and 55°C for 1 min. A final dissociation step was conducted at 60–95°C. The results were obtained using a standard curve and by analyzing the melting curve (~ 84.5°C) to approximately CT 35. Quantification of gRNA and sfRNA levels was performed as described previously [44].

In addition, IFN-α1/13 production was measured in the supernatants of infected HepG2 as recommended for sample cultures by the Human IFNA1/Interferon Alpha-1/13 ELISA Kit (RAB0541; Sigma-Aldrich).

### Analysis of IFN-stimulated response by ISRE expression in HBMEC reporter cell line

HBMEC-ISRE-Luc cells were mock-treated or infected with DENV-2 (strain 16681), L1 or L6 DENV-1, in the presence or absence of IFN-β (1000 U– 2 ng/mL; PeproTech). After 48 hpi, the cells were lysed using cell culture lysis reagent (CCLR; Promega), and the supernatants were collected after centrifugation. The luciferase activity was measured by mixing 20 μL of cell lysate with 50 μL of Luciferase Assay Reagent (Promega), and the light produced was measured using a GloMax 96 Microplate Luminometer (Promega). The results are shown in relative light units (RLUs).

### Cytokine production

Seventy-two samples of DENV-1 human sera (L1 or L6) were subjected to a selected panel to measure cytokines, chemokines, adhesion molecules and growth factors (EGF, VEGF, TNF-β, TNF-α, MIP-1β, MIP-1α, MCP-1, IP-10, IL-17, IL-15, IL-13, IL-12 (p70), IL-12 (p40), IL-10, IL-8, IL-7, IL-6, IL-5, IL-3, IL-2, IL-1RA, IL-1β, IL-1α, IFN-γ, IFN-α2, GM-CSF, G-CSF and CCL11) using the MILLIPLEX MAP Human Cytokine/Chemokine Magnetic Bead Panel–Premixed 29 Plex–Immunology Multiplex Assay (HCYTMAG-60K-PX29; Millipore) by the Luminex system in the MAGPIX instrument, according to the manufacturer’s instructions.

### Antigenicity prediction

*In silico* analyses of the putative antigenic potential of L1 and L6 lineages were performed as previously described [21]. Briefly, 20 amino acid sequences encoding the DENV-1 polyprotein were first aligned using the Multalin interface (http://multalin.toulouse.inra.fr/multalin/multalin.html) with default parameters. The consensus sequence coding for Capsid, Envelope and NS1 proteins was submitted to the BepiPred 1.0 server (http://www.cbs.dtu.dk/services/BepiPred/) for Linear B epitope prediction. The consensus sequence coding for DENV-1 polyprotein was run into the NetCTL server (http://www.cbs.dtu.dk/services/NetCTL/) to predict T-cell epitopes. Both algorithms were run using default settings. The Allele Frequency Net Database (http://www.allelefrequencies.net/) was used to select the most predominant HLA classes in the Southeast of Brazil and to set up the NetCTL server. The mean value of the epitope propensity scores for each sequence was then classified and plotted according to its potential immunogenicity.

### B and T cell activation

Eight-week-old male C57BL/6 mice were divided into 3 groups (5 animals/group) and intraperitoneally immunized with 5x10^5^ IP per mouse of DENV-1: L1 (group 1) or L6 (group 2). Mock C6/36 injections in L-15 medium (group 3) were used as controls. At 7 days p.i., the spleens from each group of mice were extracted, immersed in cold RPMI 1640 medium (Cultilab) and macerated. After centrifugation at 377 x g for 10 min, the erythrocytes were lysed in 9 mL of cold water. Lysis was stopped by adding 1 mL of 1.5 M PBS. The spleen cells were collected after centrifugation and resuspended in 1 mL of RPMI supplemented with 10% FBS (Gibco). To assess viability, an aliquot of cells was diluted 1:20 in 0.4% Trypan Blue solution (Invitrogen) and counted using a Neubauer chamber. A panel of monoclonal antibodies (Becton Dickinson, USA) specific for CD4^+^, CD8^+^ and CD21^+^ lymphocyte subsets and activation markers (CD25, CD69, CD28 and CTLA-4) was then used for cell staining (see S5 Table).

Briefly, 1x10^6^ spleen cells were distributed in 96-well polystyrene conical bottom microwell plates and centrifuged at 377 x g for 10 min. After 30 min of incubation with the antibodies at 4°C, the cells were washed twice with 0.15 M PBS and fixed in 2% paraformaldehyde in PBS. Flow cytometry acquisition of 30,000 events/tube and analysis were performed using FACSCalibur (BD Biosciences) equipment. Distinct gating strategies were used to analyze the lymphocyte subsets (CD4^+^, CD8^+^ T-cell subsets and CD21^+^ B cells) with FlowJo software. The T lymphocyte CD8^+^ and CD4^+^ subsets were selected from the CD3^+^ cell population, and B lymphocytes were selected using the CD21^+^ marker. The frequency of cells was determined using quadrant statistics. Limits for the quadrant markers were always set based on negative populations and isotype controls. To analyze the CD8^+^ lymphocytes, the quadrants were always set for CD8 high populations to avoid including CD8 low NK cells. The results were expressed as percentages of cells for the different gated lymphocyte subpopulations analyzed. The expression of activation markers was evaluated inside each lymphocyte population by measuring the mean intensity fluorescence (MIF), which represents the number of molecules per cell.

The human PBMCs (2x10^5^ cells) were cultured with L1 or L6 DENV-1 at an MOI of 1.0 for 2 h. Control cultures were performed using supernatant from noninfected C6/36 cells (mock-infected). At 48 hpi, the cells were harvested and incubated with CD38-APC, HLA-DR-FITC and CD3-Pacific Blue (eBiosciences). The frequency of CD38^+^/HLA-DR^+^ among the total PBMCs, CD4^+^ or CD8^+^ cells for the analysis of PBMC activation was determined by flow cytometry using FACSCalibur (BD Biosciences) equipment and FlowJo software. Additionally, after 12 days of culture, the supernatants were harvested and the IgM or IgG levels were analyzed by capture ELISA. Briefly, the ELISA plates were incubated with anti-human IgM or IgG antibodies at 3 mg/mL (Sigma-Aldrich) overnight at 4°C. The plates were blocked with PBS containing 10% FCS for 2 h at 37°C and washed with PBS-0.05% Tween 20 (PBS-T), and serial dilutions of the supernatant samples were added and incubated overnight at 4°C. Serial dilutions of purified human IgM or IgG were also added to generate a standard curve. The plates were incubated with alkaline phosphatase (AP)-conjugated anti-human IgM or IgG (1 mg/mL; Invitrogen) for 2 h at 37°C, washed and developed using pNPP substrate (Sigma-Aldrich). The reaction was read at 405 nm using an ELISA reader (Bio-Rad Laboratories). Also, the secretion levels of IFN-γ, IL-6 and IL-8 were evaluated by ELISA, according to manufacturer’s protocol (PeproTech).

### Statistical analysis

The comparative analysis of the two lineages was performed using Student’s T test and Chi-squared test. Fisher’s exact test was used to compare proportions, and the Mann-Whitney nonparametric test was also applied. P values ≤0.05 were considered significant. All analyses were performed using GraphPad Prism version 6.01.

### Accession numbers

The envelope sequences of the DENV-1 isolates analyzed in this study are in GenBank under the following accession numbers: KT438562, KT438564, KT438565, KT438566, KT438567, KT438568, KT438569, KT438570, KT438571, KT438572, KT438573, KT438574, KT438575, KT438576, KT438577, KT438578, KT438579, KT438581, KT438582 and KT438583. Additional genomic sequences used in the aa substitutions analysis can be found as GenBank: KP188543 and KP188540.

## Supporting information

S1 Fig Comparative quantification of growth curves of two strains of either L1 or L6 lineage infected at an MOI of 0.1 in *Ae*. *albopictus* cell line (C6/36). (A) Quantification of cells by flow cytometry-based assay. (B) Quantification of supernatants by qRT-PCR method.
(TIF)

S2 Fig Evaluation of cytokines in human leukocytes (PBMCs) cultured with L1 or L6 DENV-1 at an MOI of 1.0 and analyzed by capture ELISA. (A, B, C and D) Average and individual data levels for IFN-γ (B), IL-6 (C) and IL-8 (D) production (Wilcoxon test).
(TIF)

S3 Fig Quantification of cytokines, chemokines, adhesion molecules and growth factors that were not significantly different in L1 or L6 DENV-1-infected patients. (A) IL-1α, IL-1β, IL-2, IL-3, IL-5, IL-6, IL-10, IL-12p70, IL-15, IFN-γ, TNF- α and TNF- β. (B) IL-8, IP-10, MCP-1, MIP-1α, MIP-1β, G-CSF and GM-CSF (Mann-Whitney test).
(TIF)

S4 Fig Mosquitoes coinfection with different L1:L6 ratios. Dom Pedro mosquitoes were fed with different L1:L6 ratios. Fourteen dpi viral cDNA copy numbers were determined in the body and head of the mosquitoes by Taqman-based qPCR (Student’s T test).
(TIF)

S1 Table Names and sequences of sense and antisense primers with amplicons used in the sequencing reactions. (DOCX)

S2 Table Names and sequences of sense and antisense primers with amplicons used for genotyping. (DOCX)

S3 Table Name, identified lineage and sequence of probes used for genotyping. (DOCX)

S4 Table Names and sequences of sense and antisense primers with amplicons used for the quantification of sfRNA and gRNA levels. (DOCX)

S5 Table Monoclonal antibodies and activation markers used for immunostaining and flow cytometry analysis. (DOCX)
