# Supplementary figures and images for: Viral immunogenicity determines epidemiological fitness in a cohort of DENV-1 infection in Brazil
Source: PLoS Negl Trop Dis. 2018 May 29;12(5):e0006525. doi: 10.1371/journal.pntd.0006525 (PMC5993327; doi:10.1371/journal.pntd.0006525)

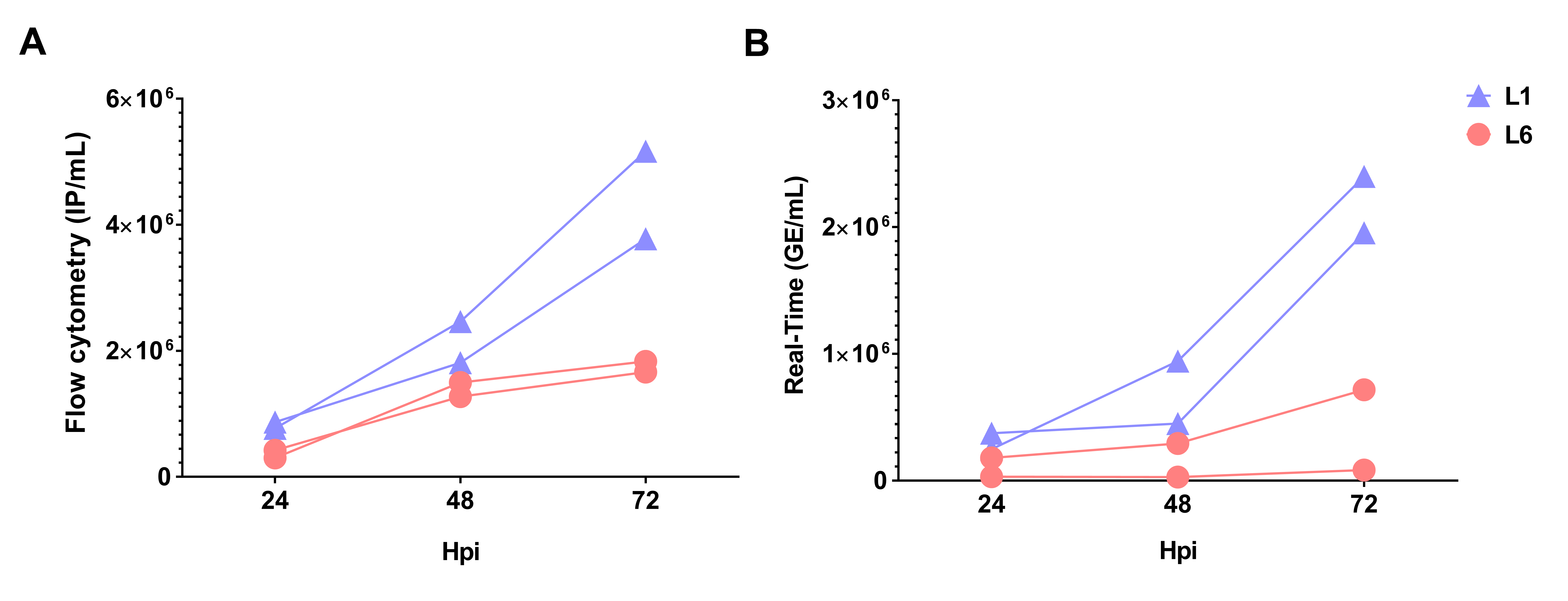

Supplement: S1 Fig — (A) Quantification of cells by flow cytometry-based assay. (B) Quantification of supernatants by qRT-PCR method. (TIF) [file pntd.0006525.s001.tif]

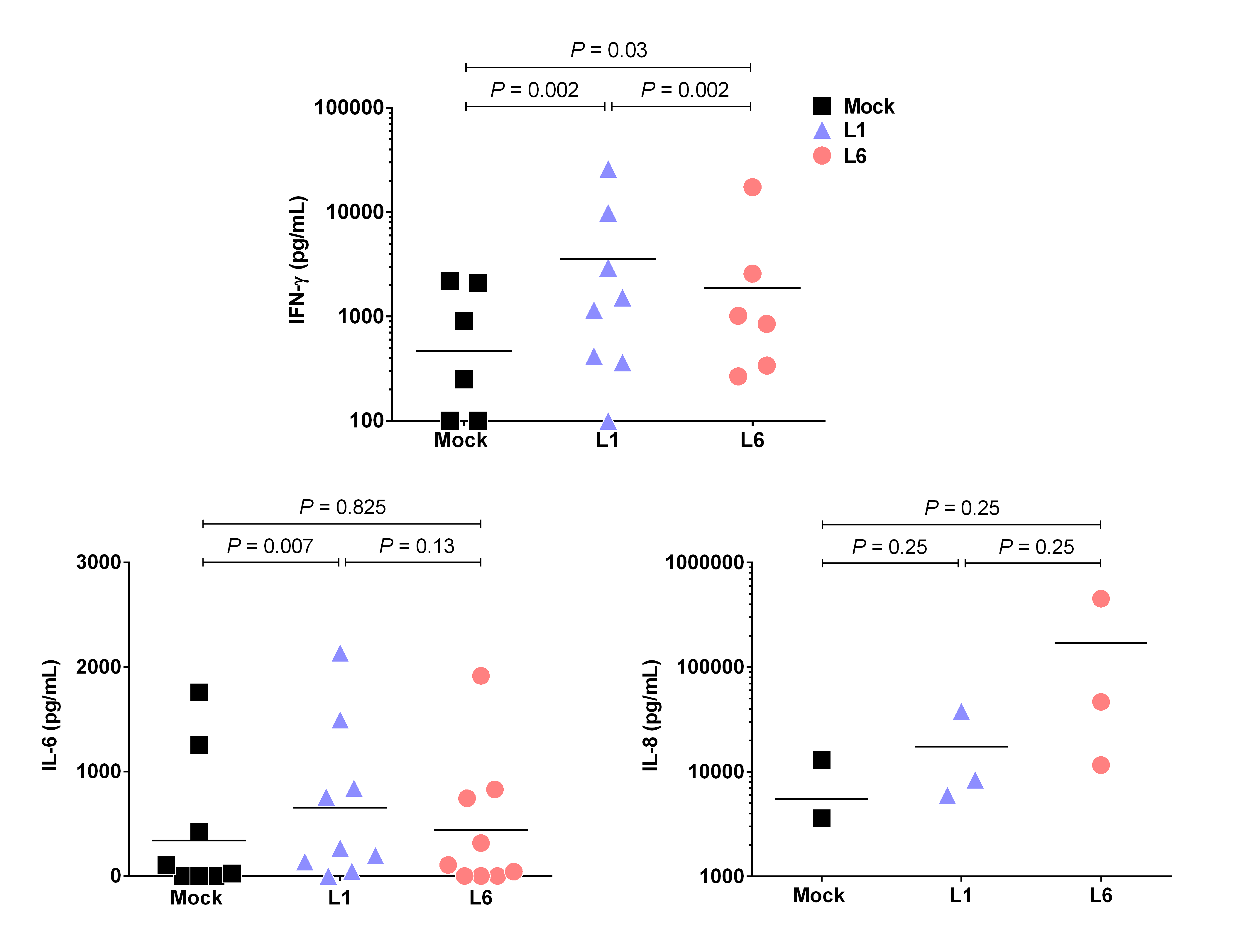

Supplement: S2 Fig — (A, B, C and D) Average and individual data levels for IFN-γ (B), IL-6 (C) and IL-8 (D) production (Wilcoxon test). (TIF) [file pntd.0006525.s002.tif]

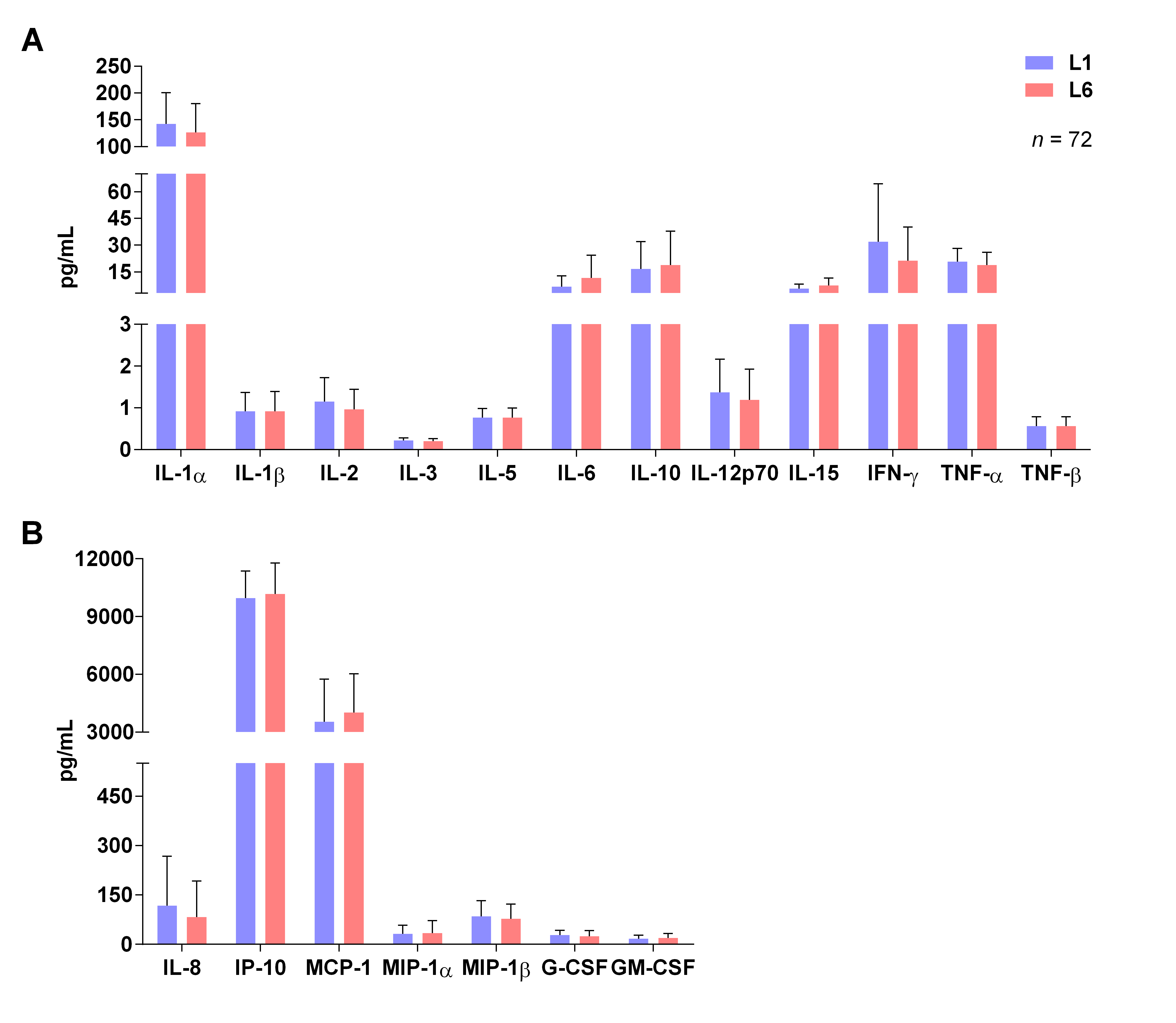

Supplement: S3 Fig — (A) IL-1α, IL-1β, IL-2, IL-3, IL-5, IL-6, IL-10, IL-12p70, IL-15, IFN-γ, TNF- α and TNF- β. (B) IL-8, IP-10, MCP-1, MIP-1α, MIP-1β, G-CSF and GM-CSF (Mann-Whitney test). (TIF) [file pntd.0006525.s003.tif]

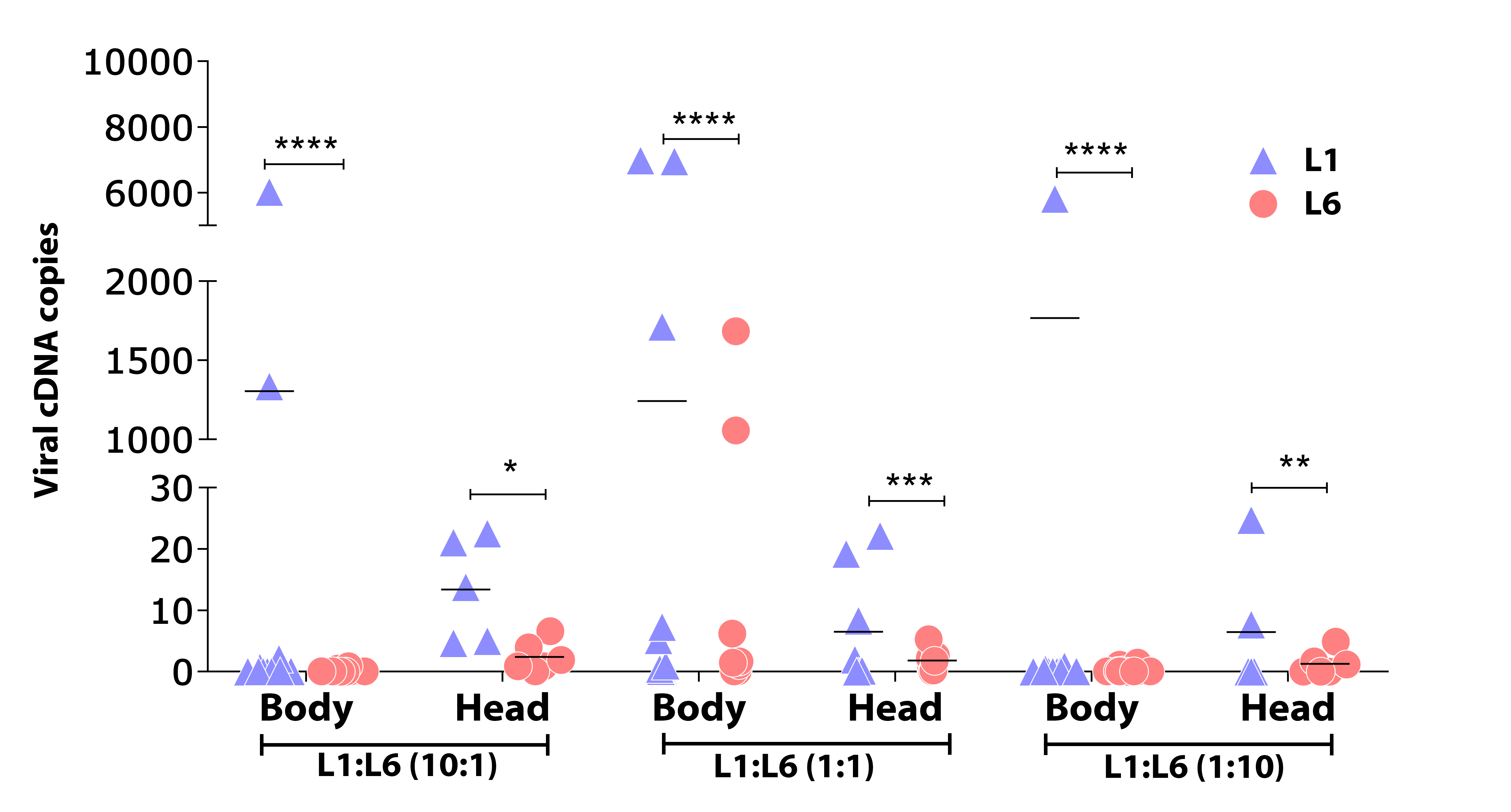

Supplement: S4 Fig — Dom Pedro mosquitoes were fed with different L1:L6 ratios. Fourteen dpi viral cDNA copy numbers were determined in the body and head of the mosquitoes by Taqman-based qPCR (Student’s T test). (TIF) [file pntd.0006525.s004.tif]
