# Supplementary material for: Viral immunogenicity determines epidemiological fitness in a cohort of DENV-1 infection in Brazil
Source: PLoS Negl Trop Dis. 2018 May 29;12(5):e0006525. doi: 10.1371/journal.pntd.0006525 (PMC5993327; doi:10.1371/journal.pntd.0006525)
Supplement: S1 Table — (DOCX) [file pntd.0006525.s005.docx]

**S1 Table.** **Names and sequences of sense and antisense primers with amplicons used in the sequencing reactions.**

| **Primers** | **Sequences (5'-3')** | **Amplicons (pb)** |
| --- | --- | --- |
| d1s3 (+) | AAA CGT TCC GTS GCA CTG GC | 1855 |
| d1s4 (+) | TGT GTG TCG MCG AAC GTT | 1355 |
| d1s5 (+) | GCA ATG CAC ACY GCG TTG | 850 |
| d1s6 (+) | GGY TCT ATA GGA GGR GTG TTC AC | 358 |
| d1a16 (-) | CAR CTT CCA RGT YTC GTT CTT | 1855 |
| d1a17 (-) | CCA ATG GCY GCT GAY AGT CT | 1855 |
| d1a18 (-) | AAA GGT GGY TCY GYY TCA AT | 1348 |
| d1a19 (-) | GTT TGT GGA CRA GCC ATG ATT | 861 |
| d1a20 (-) | CGT CTT CAA GAG TTC AAT GTC C | 374 |

(+): sense; (-): antisense.
