# Supplementary material for: Viral immunogenicity determines epidemiological fitness in a cohort of DENV-1 infection in Brazil
Source: PLoS Negl Trop Dis. 2018 May 29;12(5):e0006525. doi: 10.1371/journal.pntd.0006525 (PMC5993327; doi:10.1371/journal.pntd.0006525)
Supplement: S2 Table — (DOCX) [file pntd.0006525.s006.docx]

### **S2 Table.** **Names and sequences of sense and antisense primers with amplicons used for genotyping.**

| **Primers** | **Sequences (5'-3')** | **Amplicons (pb)** |
| --- | --- | --- |
| 2021_F (+) | GGC AGG TGA AAA AGC TTT GAA | 70 |
| 2021_R (-) | GGC AGG TGA AAA AGC TTT GAA |  |
| 8587_F (+) | GAC ACG CGC ACA CCA AGA | 69 |
| 8587_R (-) | CCC ATA ACC ACT TGG CTG TCA |  |

(+): sense; (-): antisense.
