# Supplementary material for: Viral immunogenicity determines epidemiological fitness in a cohort of DENV-1 infection in Brazil
Source: PLoS Negl Trop Dis. 2018 May 29;12(5):e0006525. doi: 10.1371/journal.pntd.0006525 (PMC5993327; doi:10.1371/journal.pntd.0006525)
Supplement: S3 Table — (DOCX) [file pntd.0006525.s007.docx]

### **S3 Table. Name, identified lineage and sequence of probes used for genotyping.**

| **Probes** | **Lineages** | **Sequences (5'-3')** |
| --- | --- | --- |
| 2021_P1 | L1 | FAM–CTG GTT CAA GAG AGG A–MGB |
| 8587_P2 | L6 | VIC–CAA AAA GAG GCA CAG CA–MGB |
