# Supplementary material for: Viral immunogenicity determines epidemiological fitness in a cohort of DENV-1 infection in Brazil
Source: PLoS Negl Trop Dis. 2018 May 29;12(5):e0006525. doi: 10.1371/journal.pntd.0006525 (PMC5993327; doi:10.1371/journal.pntd.0006525)
Supplement: S4 Table — (DOCX) [file pntd.0006525.s008.docx]

### **S4 Table. Names and sequences of sense and antisense primers with amplicons used for the quantification of sfRNA and gRNA levels.**

| **Primers** | **Sequences (5'-3')** | **Amplicons (bp)** |
| --- | --- | --- |
| D1GSF (+) | AGG CCG GAT TAA GCC ATA GT | 235 |
| D1SF (+) | CAG GCC GGA TTA AGC CAT AGT | 84 |
| D1GSR (-) | GGC TTT CGG CCT GAC TTC AT |  |

(+): sense; (-): antisense.
