# Supplementary material for: Viral immunogenicity determines epidemiological fitness in a cohort of DENV-1 infection in Brazil
Source: PLoS Negl Trop Dis. 2018 May 29;12(5):e0006525. doi: 10.1371/journal.pntd.0006525 (PMC5993327; doi:10.1371/journal.pntd.0006525)
Supplement: S5 Table — (DOCX) [file pntd.0006525.s009.docx]

**S5 Table.** Monoclonal antibodies and activation markers used for immunostaining and flow cytometry analysis.

| **Antibody** | **Fluorochrome** | **Clone** |
| --- | --- | --- |
| Anti-mouse CD3 | FITC | 145-2C11 |
| Anti-mouse CD4 | PerCP | RM4-5 |
| Anti-mouse CD8 | PerCP | 53-6.7 |
| Anti-mouse CD25 | PE | PC61 |
| Anti-mouse CD69 | PE | H1.2F3 |
| Anti-mouse CD28 | APC | 37.51 |
| Anti-mouse CTLA-4 | APC | UC10-4B9 |
